# The Human Amnion Epithelial Cell Secretome Decreases Hepatic Fibrosis in Mice with Chronic Liver Fibrosis

**DOI:** 10.3389/fphar.2017.00748

**Published:** 2017-10-24

**Authors:** Majid Alhomrani, Jeanne Correia, Marcus Zavou, Bryan Leaw, Nathan Kuk, Rong Xu, Mohamed I. Saad, Alexander Hodge, David W. Greening, Rebecca Lim, William Sievert

**Affiliations:** ^1^Centre for Inflammatory Diseases, Monash University, Clayton, VIC, Australia; ^2^Hudson Institute of Medical Research, Clayton, VIC, Australia; ^3^Medical College, Taif University, Taif, Saudi Arabia; ^4^Gastroenterology and Hepatology Unit, Monash Health, Clayton, VIC, Australia; ^5^Department of Obstetrics and Gynaecology, Monash University, Clayton, VIC, Australia; ^6^Department of Biochemistry and Genetics, La Trobe Institute for Molecular Science, La Trobe University, Melbourne, VIC, Australia; ^7^Department of Molecular and Translational Science, Monash University, Clayton, VIC, Australia

**Keywords:** human amnion epithelial cells, extracellular vesicles, liver fibrosis, macrophages, anti-fibrotic therapy, secretome, soluble-factors

## Abstract

**Background:** Hepatic stellate cells (HSCs) are the primary collagen-secreting cells in the liver. While HSCs are the major cell type involved in the pathogenesis of liver fibrosis, hepatic macrophages also play an important role in mediating fibrogenesis and fibrosis resolution. Previously, we observed a reduction in HSC activation, proliferation, and collagen synthesis following exposure to human amnion epithelial cells (hAEC) and hAEC-conditioned media (hAEC-CM). This suggested that specific factors secreted by hAEC might be effective in ameliorating liver fibrosis. hAEC-derived extracellular vesicles (hAEC-EVs), which are nanosized (40–100 nm) membrane bound vesicles, may act as novel cell–cell communicators. Accordingly, we evaluated the efficacy of hAEC-EV in modulating liver fibrosis in a mouse model of chronic liver fibrosis and in human HSC.

**Methods:** The hAEC-EVs were isolated and characterized. C57BL/6 mice with CCl_4_-induced liver fibrosis were administered hAEC-EV, hAEC-CM, or hAEC-EV depleted medium (hAEC-EVDM). LX2 cells, a human HSC line, and bone marrow-derived mouse macrophages were exposed to hAEC-EV, hAEC-CM, and hAEC-EVDM. Mass spectrometry was used to examine the proteome profile of each preparation.

**Results:** The extent of liver fibrosis and number of activated HSCs were reduced significantly in CCl_4_-treated mice given hAEC-EVs, hAEC-CM, and hAEC EVDM compared to untreated controls. Hepatic macrophages were significantly decreased in all treatment groups, where a predominant M2 phenotype was observed. Human HSCs cultured with hAEC-EV and hAEC-CM displayed a significant reduction in collagen synthesis and hAEC-EV, hAEC-CM, and hAEC-EVDM altered macrophage polarization in bone marrow-derived mouse macrophages. Proteome analysis showed that 164 proteins were unique to hAEC-EV in comparison to hAEC-CM and hAEC-EVDM, and 51 proteins were co-identified components with the hAEC-EV fraction.

**Conclusion:** This study provides novel data showing that hAEC-derived EVs significantly reduced liver fibrosis and macrophage infiltration to an extent similar to hAEC-EVDM and hAEC-CM. hAEC-EV-based therapy may be a potential therapeutic option for liver fibrosis.

## Introduction

Hepatic fibrosis results from chronic inflammatory liver injury attributed to many factors including steatohepatitis, viral hepatitis, alcohol, toxins, and autoimmune disease. Chronic liver injury may result in a dysregulated wound-healing response with persistent infiltration of inflammatory cells and accumulation of extracellular matrix (ECM) due to pathogenic activation of quiescent hepatic stellate cells (HSCs) with subsequent phenotypic transformation into collagen-secreting myofibroblasts. If this response is persistent, normal hepatic architecture is altered by extensive fibrosis and loss of functional hepatocyte mass leading to cirrhosis and portal hypertension. Patients with cirrhosis are at increased risk of liver failure and hepatocellular carcinoma (HCC) (Liedtke et al., 2013). Currently, the only effective therapy for patients with end-stage liver disease is liver transplantation, a complex surgical procedure reliant on donor availability (Francoz et al., 2007). The complexity of the procedure, an increase in patients requiring liver transplantation and donor shortages, demonstrates the urgent need for an alternate therapy.

Stem cells have been investigated as a potential therapy to treat end-stage liver disease. Mesenchymal stromal cells (MSC) have shown encouraging preclinical results in animal models of liver fibrosis (Chang et al., 2009; Cho et al., 2011; Huang et al., 2013) and in human clinical trials (Amin et al., 2013; Mohamadnejad et al., 2013). However, there are several arguments against the clinical use of MSC, including the possibility of malignant transformation and the requirement for *in vitro* expansion prior to clinical use (Baglio et al., 2012).

The human placenta contains several types of stem and stem-like cells including human amnion epithelial cells (hAEC). hAEC are non-tumorigenic and due to their low expression of HLA-Class IA and absent expression of Class II molecules do not induce host immune rejection (Wolbank et al., 2007; Pratama et al., 2011). hAEC are highly abundant – approximately 150 × 10^6^ cells can be isolated from a single-term amniotic membrane, which is adequate for clinical use without the need for *in vitro* expansion (Murphy et al., 2010). We and others have shown the anti-fibrotic effect of hAEC in mouse models of bleomycin-induced lung fibrosis (Murphy et al., 2011), and in both acute (Manuelpillai et al., 2010) and chronic (Manuelpillai et al., 2012) liver fibrosis induced by carbon tetrachloride (CCl_4_). Moreover, studies have shown that hAEC have low rates of engraftment in injured tissue (Vosdoganes et al., 2011, 2013), which suggests that hAEC mediate their effects through release of paracrine factors. We have shown that hAEC-conditioned media (hAEC-CM) contains soluble factors which suppress proliferation, activation, and collagen production and induce apoptosis of human HSC (Hodge et al., 2014).

Evidence that stem cell conditioned media alone could exert therapeutic effects has given rise to a new theory on the mechanisms of certain cell therapies. For example, the MSC secretome is thought to be responsible for many of its physiological functions (Makridakis et al., 2013; Lee S.K. et al., 2015). More recently, crucial evidence has come to light suggesting that extracellular vesicles (EVs) are the effectors of MSC paracrine actions (Baglio et al., 2012). EVs are complex membrane enclosed nanoparticles that carry a cargo of select proteins, RNAs, and lipids (Xu et al., 2016). They are categorized by their size and biogenesis and include microvesicles (100–1000 nm), apoptotic vesicles (20–1500 nm), ectosomes (50–200 nm), and exosomes (40–150 nm) (Xu et al., 2016). However, EVs derived from hAEC (hAEC-EV) have yet to be characterized and as such their ability to reduce fibrosis following transplantation remains unknown. In this study, we isolated hAEC-EV by serial ultracentrifugation, performed in-depth characterization of isolated EVs and their protein cargo, and investigated the therapeutic efficacy of hAEC-EV in reducing hepatic fibrosis.

## Materials and Methods

### Isolation of hAEC

Human amnion epithelial cells (hAEC) were isolated from the placentas of 16 healthy women undergoing cesarean section at term (37–40 weeks gestation) as described previously (Murphy et al., 2010; Xu et al., 2016). Written informed consent was obtained from each woman. The study was approved by the Monash Health Human Research Ethics Committee (approval number: 01067B).

### hAEC-Conditioned Media (CM)

The hAEC-CM was prepared by culturing 10 million hAEC in chemically defined, serum-free Ultraculture medium (Lonza, Walkersville, MD, United States). Cultures were maintained for 4 days at 37°C in a humidified chamber containing 5% CO_2_ prior to harvesting conditioned media.

### Isolation of hAEC-EV and Extracellular Vesicle Depleted Media (EVDM)

Serial centrifugation was used to obtain EV and EVDM from collected hAEC-CM (Ng et al., 2013). Briefly, hAEC-CM was centrifuged at 300 g for 10 min at 4°C and at 2000 g for 10 min at 4°C to remove cells and cellular debris. The supernatant then was collected and centrifuged at 10,000 g for 30 min at 4°C to remove large shed microvesicles (Meldolesi, 2015). The supernatant was subjected to ultracentrifugation 110,000 g for 90 min at 4°C (KQ424, Optima L-90K Ultracentrifuge, Beckman, Australia). The supernatant (EV-depleted media, EVDM) was collected and the pellet was washed by resuspending in PBS and underwent a final ultracentrifugation step at 110,000 g for 90 min at 4°C. The washed EV pellet was then resuspended in PBS and stored in aliquots. All hAEC components, EV, CM, and EVDM, were stored at -80°C until required.

### Immunoblotting

The EVDM was concentrated using a membrane with a 100 Kda molecular weight cutoff (UFC710008, Merck, Australia). Proteins were separated on a NuPAGE 4–12% Bis–Tris Precast gel (Thermo Fisher Scientific, Australia) and transferred to nitrocellulose membrane (Thermo Fisher Scientific). Membranes were incubated with mouse anti-human Alix (3A9, Abcam, Cambridge, MA, United States, 1/1000), mouse anti-human CD81 (M38, Thermo Fisher Scientific, 1/250), and mouse anti-human CD63 (TS63, Thermo Fisher Scientific, 1/250). Protein bands were detected using Odyssey imaging system (LI-Cor, Lincoln, NE, United States).

### Transmission Electron Microscopy

Extracellular vesicles (EVs) suspended in PBS were placed on a formvar-carbon-coated electron microscope grid for 20 min then fixed in 1% glutaraldehyde for 5 min. Grids were then placed in uranyl-oxalate solution (UOA) followed by methylose–cellulose. These were then thoroughly dried before being subjected to a scanning electron microscope (H7500, Hitachi, Japan) at 70 kV.

### Nanoparticle Tracking Analysis

The diameter and concentration of vesicles were determined using a NanoSight NS300 system (NanoSight technology, Malvern, United Kingdom) equipped with a blue laser (488 nm). Briefly, EVs and EVDM loaded into a flow-cell top plate using a syringe pump. Three videos (1 min) were recorded for each sample, merged and analyzed by NTA software (Build 3.1.45).

### Animals

A male C57Bl/6J mice of 6- to 8-weeks-old were purchased from Monash Animal Services, Melbourne, VIC, Australia and maintained in pathogen-free conditions at the Monash Medical Centre Animal Facility. Twelve hourly dark–light cycles were maintained with food and water access provided ad libitum. The Monash University Animal Ethics Committee approved all animal experiments and mice received care under the Australian Code of Practice for the care and use of animals for scientific purposes.

### CCl_4_, CM, EVDM, and EV Administration

Mice were divided into 5 groups (*n* = 6–8) and, other than the healthy untreated group, each group received intraperitoneal injections (IP) of carbon tetrachloride (CCl_4_) twice weekly for 12 weeks at 1 μL/g body weight, diluted 1:10 in olive oil as previously described (Manuelpillai et al., 2012). Eight weeks later, when bridging fibrosis was evident, mice were administered three intravenous doses of either 350 μL CM, 350 μL EVDM (∼2 × 10^6^ particles), or 1 μg EV (∼24 × 10^6^ particles) in 350 μL saline or saline only (as controls) weekly for the last 4 weeks. All mice were culled at week 12 after commencing CCl_4_ administration and blood and liver tissue were collected.

### Picrosirius Red Staining

Mouse livers were fixed in 10% neutral buffered formalin and were cut into 4-μm sections. The sections were dewaxed and rehydrated and incubated for 90 min in Picrosirius red (Direct Red 80, 0.1% wt/vol in saturated picric acid, Sigma–Aldrich, St. Louis, MO, United States), then washed with acetic acid and water (1:200) and mounted in DPX (Sigma–Aldrich, St. Louis, MO, United States). Five non-overlapping fields were acquired from untreated mice (*n* = 8), and mice treated with hAEC-CM (*n* = 8), EVDM (*n* = 6), and EV (*n* = 8). Fibrosis area was measured with ImageJ software package (NIH Image, Bethesda, MD, United States).

### Immunohistochemistry

Four-micron-thick paraffin sections of liver tissue from untreated and treated mice as described above were dewaxed and rehydrated and heat-mediated antigen retrieval performed by incubation with 10 mM sodium citrate (pH 6). Sections were then incubated with 0.3% (v/v) H_2_O_2_ for 15 min and blocked with a universal protein blocking solution for 1 h. Primary antibodies F4/80 (MCA497, Bio-Rad, Puchheim, Germany, 1:600) and α-Smooth Muscle Actin (α-SMA) (A5228-200UL, Sigma–Aldrich, St. Louis, MO, United States, 1:1500) were applied and the tissue sections were incubated in a humidified chamber overnight at 4°C or 30 min at room temperature, respectively. The sections were then washed and biotinylated rabbit anti-mouse IgG2a (E0464, Dako, Carpinteria, CA, United States, 1:500,) and rabbit anti-rat IgG (E0468, Dako, Carpinteria, CA, United States, 1:150) were applied for 1 h at room temperature followed by visualization using the Vectastain ABC HRP kit (Vector Laboratories, Burlingame, CA, United States) and DAB substrate (Dako, Carpinteria, CA, United States).

### Immunofluorescent Staining

Serial 4-μm paraffin-embedded sections from untreated and treated mice as described above were dewaxed, rehydrated, and incubated in 10 mM sodium citrate pH 6 for heat-mediated antigen retrieval. To remove auto-fluorescence, Sudan Black was applied to the tissue sections for 15 min. Prior to immunolabeling, tissues were blocked with a universal protein blocking solution for 1 h and incubated with primary antibodies Rat F4/80 (MCA497, Bio-Rad, Puchheim, Germany, 1:100) and rabbit CD86 (EP1158Y, Novus Biological, Littleton, CO, United States 1:300) or rabbit CD206 (Ab64693, Abcam, Cambridge, MA, United States 1:500). Tissue sections were then washed three times and incubated with secondary antibodies (Alexa Fluor conjugates, Life Technologies, Frederick, MD, United States) including goat anti-rat 488 (1:100), donkey anti-rabbit 568 (1:100), and goat anti-rabbit 647 (1:500) for 90 min followed by incubation with DAPI (Sigma–Aldrich, St. Louis, MO, United States) for 10 min and mounted using fluorescent mounting medium (Dako, Carpinteria, CA, United States).

### *In Vitro* Effects of hAEC-EV on Macrophage Polarization

Immortalized mouse bone marrow macrophages from wild-type mice (a gift from Associate Professor Ashley Mansell, Centre for Innate Immunity and Infectious Diseases, Hudson Institute of Medical Research) were plated on 6-well plates and cultured in Dulbecco’s modified Eagle’s medium (DMEM) with high glucose (DMEM/F12-High Glucose, Life Technologies, Frederick, MD, United States), and antibiotics (50 U/ml Penicillin and 50 μg/ml streptomycin, Life Technologies, Frederick, MD, United States). On day 1, M1 activation was assessed following cell stimulation using lipopolysaccharide (LPS; 10 ng/ml) and interferon-γ (IFN-γ; 10 ng/ml) (Life Technologies, Frederick, MD, United States), while M2 activation was assessed using interleukins-4 and interleukins-13 (PeproTech, Rocky Hill, NJ, United States) (10 ng/ml each) (Bansal et al., 2015). To investigate their effect on macrophage polarization, 10 μg hAEC-EV, 50% hAEC-CM, or 50% hAEC-EVDM obtained from three donors (*n* = 3) were added the next day to M1, M2, and naïve macrophage cultures. After 24 h, cells were collected and then stained for the M1 marker CD86 (V450, 1:200, BD Biosciences, San Jose, CA, United States) and M2 marker CD206 (Alexa Fluor 647, 1:200, BioLegend, San Diego, CA, United States). Samples were analyzed by flow cytometry using a FACS Canto II machine (Becton-Dickinson). These experiments were performed in duplicate.

### Collagen Synthesis

Hepatic stellate cell (HSC) collagen synthesis was analyzed as described earlier (Hodge et al., 2014). Briefly, human immortalized HSCs (LX2 cell line, a kind gift of Professor Scott Friedman, NY, United States) were serum starved in DMEM containing 5% FBS followed by culture in Ultraculture media overnight at 37°C. In the treatment groups, cells were cultured in 50% Ultraculture media and 50% hAEC-CM, 50% hAEC-EVDM, or 50% PBS with 10 μg EV. In the control groups, HSCs were cultured either in 100% Ultraculture media as a control for CM and EVDM or 50% Ultraculture media and 50% PBS as a control for EV. [^3^H] Proline (1 μCi, PerkinElmer, Boston, MA, United States) was added to each sample.

### Enzyme-Linked Immunosorbent Assay

Snap-frozen liver tissue was homogenized in lysis buffer (50 mM Tris–HCl, 150 mM NaCl, 1 mM EDTA, 1% Triton X-100, 0.5% Tween-20, 0.1% SDS) containing a protease inhibitor cocktail (Roche, Mannheim, Germany). TGF-β was measured by ELISA (R&D Systems, Minneapolis, MN, United States) according to manufacturer’s instructions. The data were normalized against total protein concentration measured using a bicinchoninic acid (BCA) assay (Pierce BCA Protein Assay Kit, Thermo Fisher Scientific, United States).

### Image Quantification and Analysis

Sirius red, F4/80, and α-SMA immunostaining were quantified in five non-overlapping fields of view per animal using a Olympus BX41 upright microscope at 10× magnification. A mean of means was calculated for each experimental group using the threshold function in the ImageJ software package (NIH Image, Bethesda, MD, United States). Data are represented as percentage (%) of positive area per field. M1 and M2 macrophages were identified as F4/80^+^/CD86^+^ and F4/80^+^/CD206^+^, respectively, in five non-overlapping fields of view and normalized to the number of DAPI^+^ cells using an Olympus FV1200 confocal microscope at 10× magnification. We carried out negative controls in the absence of primary antibodies for all stains to indicate the level of background.

### Proteomic Analysis

A total of 30 μg pooled EVs, CM, and EVDM isolated from 10 amnions were analyzed by mass-spectrometry-based proteomics using an in-solution digestion approach followed by nanoliquid chromatography (Ultimate 3000 RSLCnano) coupled directly to a Q-Exactive HF Orbitrap (Thermo Fisher Scientific) mass spectrometer (MS) operated in data-dependent acquisition mode with technical duplicates. Peptides were loaded (Acclaim PepMap100, 5 mm × 300 μm i.d., μ-Precolumn packed with 5 μm C18 beads, Thermo Fisher Scientific) and separated (BioSphere C18 1.9 μm 120Å, 360/75 μm × 400 mm, NanoSeparations) with a 120-min gradient from 2 to 100% (v/v) phase B, 0.1% (v/v) FA in 80% (v/v) acetonitrile (ACN), 2–100% 0.1% FA in ACN, 2–40% from 0 to 100 min, and 40–80% from 100 to 110 min at a flow rate of 250 nL/min operated at 55°C.

The mass spectrometer (MS) was operated in data-dependent mode where the top 10 most abundant precursor ions in the survey scan (350–1500 Th) were selected for MS/MS fragmentation. Survey scans were acquired at a resolution of 60,000, with MS/MS resolution of 15,000. Unassigned precursor ion charge states and singly charged species were rejected, and peptide match disabled. The isolation window was set to 1.4 Th and selected precursors fragmented by HCD with normalized collision energies of 25 with a maximum ion injection time of 110 ms. Ion target values were set to 3e6 and 1e5 for survey and MS/MS scans, respectively. Dynamic exclusion was activated for 30 s. Data were acquired using Xcalibur software v4.0 (Thermo Fisher Scientific).

### Database Searching and Protein Identification

Raw data were preprocessed as described (Gorshkov et al., 2015) and processed using MaxQuant (Cox and Mann, 2008) (v1.5.8.3) with Andromeda (v1.5.6) using a Human-only (UniProt #133,798 entries) sequence database (March 2017). Data were searched as described (Gopal et al., 2015; Greening et al., 2016b) with a parent tolerance of 10 ppm, fragment tolerance of 0.5 Da, and minimum peptide length 7, with false discovery rate 1% at the peptide and protein levels, with peptide lists generated from a tryptic digestion with up to two missed cleavages, cysteine carbamidomethylation as fixed modification, and methionine oxidation and protein N-terminal acetylation as variable modifications (Luber et al., 2010). Contaminants and reverse identification were excluded from further data analysis. For pathway analyses, Kyoto Encyclopedia of Genes and Genomes (KEGG) and NIH Database for Annotation, Visualization and Integrated Discovery Bioinformatics Resources 6.7 (DAVID) resources were utilized using recommended analytical parameters (Huang da et al., 2009). For gene ontology enrichment and network analyses, UniProt^1^ database resource (biological process, molecular function) was utilized.

### Statistics

Data were analyzed using GraphPad Prism version 6.0 software for Mac OSX (GraphPad Software, San Diego, CA, United States). Multiple comparisons between different groups were analyzed by one-way ANOVA with *post hoc* Bonferroni correction. An unpaired *t*-test was performed to compare between control and EV in the *in vitro* collagen synthesis experiment. Data are shown as mean ± SEM. Differences were considered significant at *P* < 0.05.

## Results

### Characterization of hAEC-EV

**FIGURE 1:**
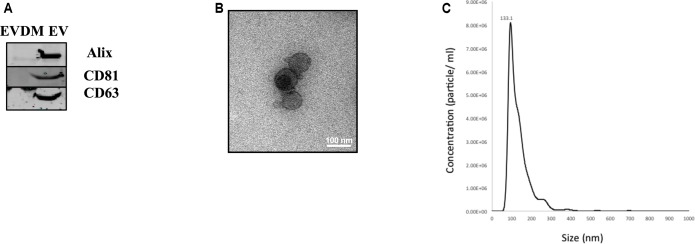
Characteristics of amnion epithelial cell–derived exosomes. **(A)** Representative Western blot images of hAEC-EV and hAEC-EVDM showing presence and enrichment of Alix, CD81 and CD63 relative to EVDM. **(B)** Electron microscopy showing cup-shaped morphology of exosomes. **(C)** Size distribution of hAEC-EVs obtained by NTA.

Extracellular vesicles released by hAEC (hAEC-EV) were prepared using serial ultracentrifugation as described previously (Ng et al., 2013). Western blot analysis showed that hAEC-EV expressed specific exosome markers including Alix, CD81, and CD63, which were absent in hAEC-EVDM (**Figure 1A**). Transmission electron microscopy showed that hAEC-EV displayed cup-shaped morphology and had a size of approximately 40–100 nm (**Figure 1B**). Nanoparticle tracking analysis was used to determine size distribution of EVs, which displayed a mean 133.1 nm diameter (**Figure 1C**). These results showed that hAEC-EV displayed the minimal criteria of exosomes and were absent in hAEC-EVDM (Lötvall et al., 2014). However, the presence of EVs in the EVDM could not be excluded completely by nanoparticle tracking analysis (**Supplementary Figure S1**).

### hAEC-CM, EVDM, and EV Suppress Hepatic Fibrosis and Reduce HSC Activation *in Vivo*

**FIGURE 2:**
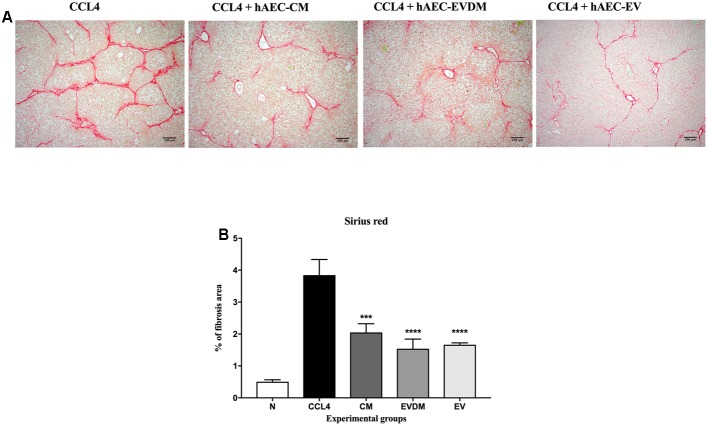
**(A)** Representative images showing collagen staining with Sirius red in liver sections from control and treated groups. Scale bar = 200 μm, 10× magnification. **(B)** The CM-, EVDM-, and EV-treated mice had significantly reduced fibrosis area compare to CCl_4_ only. Data are represented as mean ± SEM. *n* = 6–8 per group, ^∗^*P* < 0.05, ^∗∗^*P* < 0.01, ^∗∗∗^*P* < 0.001, ^∗∗∗∗^*P* < 0.0001.

**FIGURE 3:**
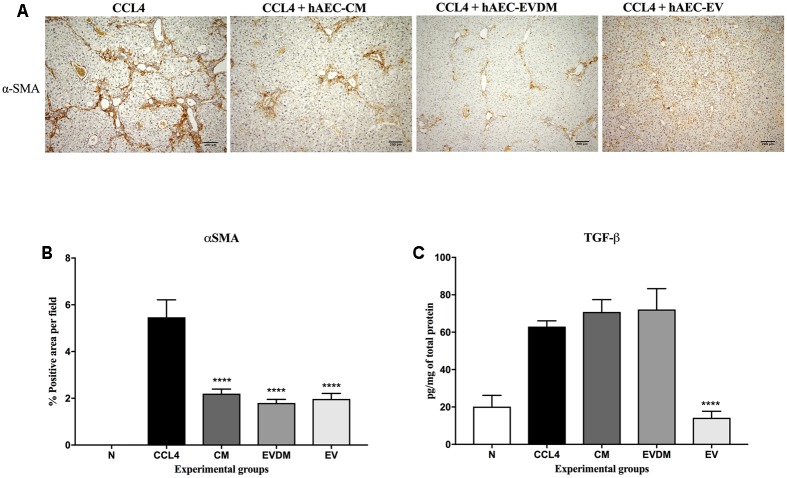
**(A)** Representative images of activated HSC stained with α-SMA from control and treated groups. Scale bar = 200 μm, 10× magnification. **(B)** The percentage of HSC in the liver was significantly decreased in mice treated with CM, EVDM, and EV compared to CCl_4_ only. **(C)** Concentration of TGF-β1 in liver was determined by ELISA and found to be significantly lower in mice treated with EV only. The data are represented as mean ± SEM. *n* = 6–8 per group, ^∗^*P* < 0.05, ^∗∗^*P* < 0.01, ^∗∗∗^*P* < 0.001, ^∗∗∗∗^*P* < 0.0001.

Mouse liver sections were stained with Picrosirius red to quantify the extent of liver fibrosis (**Figure 2A**). We observed a substantial reduction of liver fibrosis in mice given hAEC-EV (1.66 ± 0.06%, *P* < 0.0001), hAEC-CM (2.04 ± 0.27%, *P* < 0.001), and hAEC-EVDM (1.54 ± 0.30%, *P* < 0.0001), compared to mice given CCl_4_ only (3.84 ± 0.48%) (**Figure 2B**). The activation and transformation of HSC to myofibroblasts that express α-SMA typically results in increased collagen production. Fittingly, the α-SMA staining of HSC was consistent with the Picrosirius staining showing reduced hepatic fibrosis (**Figure 3A**). We noted a significant reduction in HSC number in mice given hAEC-EV (1.97 ± 0.23%, *P* < 0.0001), hAEC-CM (2.20 ± 0.19%, *P* < 0.0001), and hAEC-EVDM (1.80 ± 0.15%, *P* < 0.0001) compared to those given CCl_4_ only (5.46 ± 0.74%) (**Figure 3B**). TGF-β is a potent pro-fibrotic cytokine that initiates HSC activation (Pradere et al., 2013). We measured TGF-β1 in liver lysates by ELISA normalized to total protein content. A significant reduction in TGF-β1 was only observed in mice treated with hAEC-EV (14.22 ± 3.50 pg/mg, *P* < 0.0001), compared to control mice (63.00 ± 3.07 pg/mg). There was no significant reduction in liver lysate TGF-β1 following treatment with hAEC-CM (70.84 ± 6.59) and hAEC-EVDM (72.14 ± 11.14 pg/mg) (**Figure 3C**).

### hAEC-CM and EV Inhibit Collagen Production *in Vitro*

**FIGURE 4:**
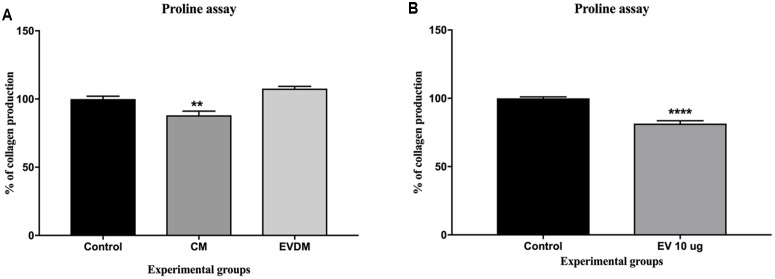
Effect of hAEC-CM, EVDM, and EV on collagen synthesis. Confluent LX2 cells were cultured with hAEC-CM, EVDM, and EV with media containing 1μCi [3H] Proline. Collagen synthesis declined significantly in LX2 exposed to CM **(A)** and EV **(B)** compared to untreated control. Results are shown as mean ± SEM. Each analysis was based on biological replicates of EV, CM, and EVDM obtained from different hAEC, ^∗^*P* < 0.05, ^∗∗^*P* < 0.01, ^∗∗∗^*P* < 0.001, ^∗∗∗∗^*P* < 0.0001.

To investigate whether hAEC-EV have a direct effect on HSC collagen production, we quantified collagen production by human stellate cells (LX2 cell line) using [^3^H] proline incorporation. Collagen production was reduced in LX2 cells treated with hAEC-EV 10 μg (81.48 ± 2.06%, *P* < 0.0001) and hAEC-CM (88.12.57 ± 3.03%, *P* < 0.01) compared to 100% Ultraculture media controls (100 ± 2.08%) (**Figure 4A**) and 50% PBS and 50% Ultraculture media controls (100 ± 1.06) (**Figure 4B**).

### hAEC-CM, EVDM, and EV Reduce Macrophage Infiltration and Induce a M2 Macrophage Phenotype

**FIGURE 5:**
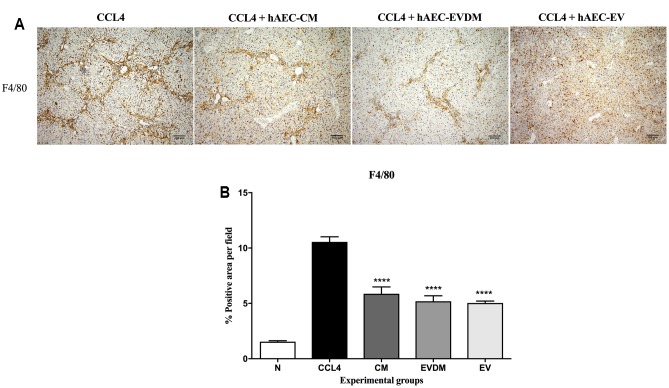
**(A)** Hepatic macrophages were identified by F4/80 immunohistochemistry. Mice with established fibrosis treated with CM, EVDM, and EV had significantly lower percentage of F4/80 macrophages in the liver compare to CCl_4_ only. Scale bar = 200 μm, 10× magnification. **(B)** Quantification of liver macrophage density using ImageJ software. The data are represented as mean ± SEM. *n* = 6–8 per group, ^∗^*P* < 0.05, ^∗∗^*P* < 0.01, ^∗∗∗^*P* < 0.001, ^∗∗∗∗^*P* < 0.0001.

**FIGURE 6:**
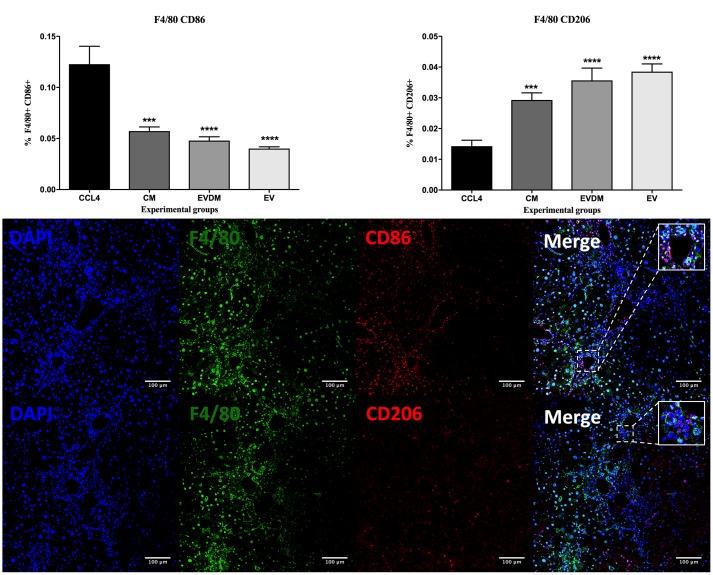
Effect of hAEC-CM, EVDM, and EV on macrophage polarization in CCL_4_-induced liver fibrosis. Quantification of F4/80^+^CD86^+^ and F4/80^+^ CD206^+^ double-labeled cells from CCl_4_ groups. Representative images of F4/80^+^CD86^+^ and F4/80^+^ CD206^+^ double-labeled cells. Scale bar = 100 μm, 20× magnification. The data are represented as mean ± SEM. *n* = 6–8 per group, ^∗^*P* < 0.05, ^∗∗^*P* < 0.01, ^∗∗∗^*P* < 0.001, ^∗∗∗∗^*P* < 0.0001.

Hepatic macrophages contribute to fibrosis progression and to fibrosis resolution. A dramatic decrease in the percentage of F4/80+ liver infiltrating macrophages was seen in mice administered hAEC-EV (5.03 ± 0.17%, *P* < 0.0001), hAEC-CM (5.87 ± 0.61%, *P* < 0.0001), and hAEC-EVDM (5.18 ± 0.50%, *P* < 0.0001) compared to those given CCl_4_ only (10.55 ± 0.46%) (**Figures 5A,B**). Polarization of macrophages toward an M2 phenotype is associated with fibrosis resolution (Manuelpillai et al., 2012). Thus, we analyzed the expression of M1 (CD86) and M2 (CD206) markers. Interestingly, the density of M1 polarized macrophages (identified by co-localization of F4/80 and CD86) was significantly higher in CCl_4_ groups (0.122 ± 0.017%) while M2 macrophage density (F4/80^+^CD206^+^) increased dramatically in hAEC-EV (0.04 ± 0.01%, *P* < 0.003), hAEC-CM (0.029 ± 0.01%, *P* < 0.002), and hAEC-EVDM-treated mice (0.04 ± 0.004%, *P* < 0.0001) (**Figure 6**).

**FIGURE 7:**
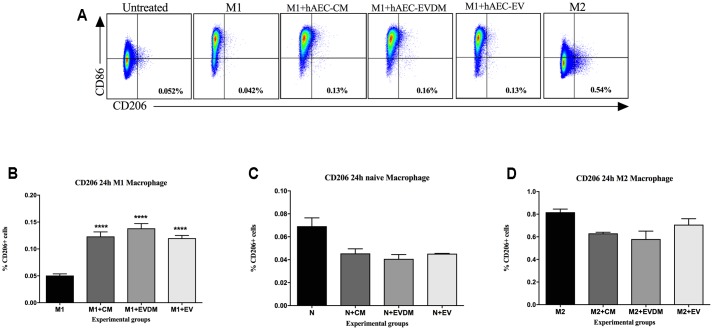
**(A)** Representative FACS plot of macrophages at 24-h post treatment. Cells were pre-gated based on size and viability. Quantification of the percentage of total number of cells gated from the FACS plot of **(B)** untreated M1-differentiated (LPS/INF-γ) macrophages or treated with hAEC-CM, EVDM, and EV, **(C)** untreated macrophages or treated with hAEC-CM, EVDM, and EV and **(D)** untreated M2-differentiated macrophages (IL-4/IL-13) or treated with hAEC-CM, EVDM, and EV. M1 and M2 differentiations were determined by CD86 and CD206, respectively. The data are represented as mean ± SEM. Each analysis was based on biological replicates of hAEC-EV, CM, and EVDM obtained from different hAEC, *n* = 3; ^∗^*P* < 0.05, ^∗∗^*P* < 0.01, ^∗∗∗^*P* < 0.001, ^∗∗∗∗^*P* < 0.0001.

To confirm that hAEC-EV, hAEC-CM, and hAEC-EVDM have a direct effect on macrophage polarization, we co-cultured bone marrow-derived macrophages with each treatment and assessed the effects of co-culture on M1 and M2 activation. We found that M2 macrophage polarization (**Figure 7A**) significantly increased after co-culture with hAEC-CM (0.12 ± 0.01%, *P* < 0.0001), hAEC-EVDM (0.14 ± 0.01%, *P* < 0.0001), and hAEC-EV at 10 μg (0.12 ± 0.01%, *P* < 0.0001) compared to M1 alone (0.05 ± 0.01) (**Figure 7B**). None of the treatments altered the phenotypes of naïve macrophages (**Figure 7C**) or M2 macrophages (**Figure 7D**).

### Proteomics Analysis of hAEC-EV, hAEC-CM, and hAEC-EVDM

**FIGURE 8:**
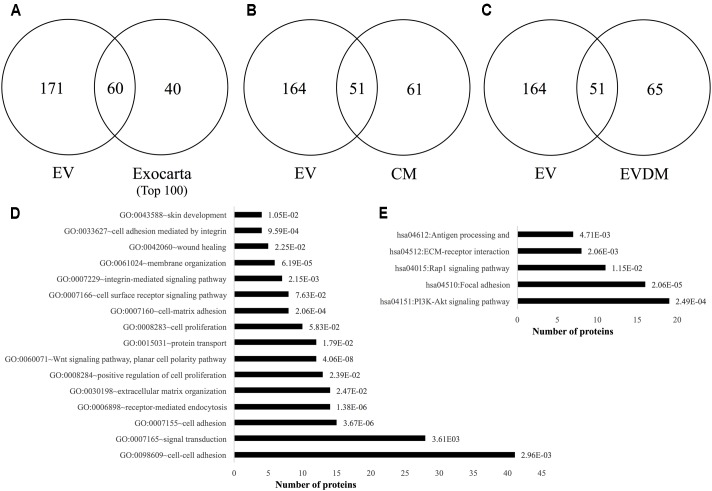
Two-way Venn diagrams of proteins distributed between hAEC-EV cargo and **(A)** ExoCarta (top 100 highly expressed exosome marker proteins) (Supplementary Table S1), **(B)** hAEC-CM (Supplementary Table S5), and **(C)** hAEC-EVDM (Supplementary Table S6). Proteomic profiling of EV cargo reveals **(D)** Gene ontology (biological process)–related function analysis with *p*-values indicated (Supplementary Table S7) and **(E)** KEGG pathway analysis with *p*-values indicated (Supplementary Table S8).

We next compared the proteome profiles of the hAEC-EV, hAEC-CM, and hAEC-EVDM using GeLC-MS/MS (Tauro et al., 2012a,b; Greening et al., 2016a, 2017). For purified EVs, this resulted in 231 proteins identified (Supplementary Table S1). We observed an abundance of typical exosome associated proteins such as tetraspanins CD9 and CD81, various Rab GTPases, and select components associated with vesicle sorting/trafficking including ARF1, LAMP1, and CLTC (Supplementary Table S1). We found 61 proteins identified in the exosome database ExoCarta (top 100 highly expressed proteins in exosomes)^2^. This supports the enrichment of select exosome marker proteins including HSPA8, CLTN, and integrins ITGA6 and ITGB1 (**Figure 8A** and Supplementary Table S2). In comparison with hAEC-CM (**Figure 8B**) and hAEC-EVDM (**Figure 8C**), we found 51 components in common with the isolated EV fraction (Supplementary Table S1). Additionally, 164 proteins were unique to hAEC-EV in comparison to hAEC-CM (Supplementary Table S3) and hAEC-EVDM (Supplementary Table S4). EV components included Milk fat globule epidermal growth factor–factor 8 (MFGE8), heat shock 72 kDa protein (HSP72), and Superoxide dismutase Cu-Zn SOD (SOD1). Furthermore, we found 61 proteins unique to hAEC-CM (Supplementary Table S5) and 65 proteins unique to hAEC-EVDM (Supplementary Table S6).

When subjected to a gene ontology analysis, we observed enrichment of biological processes associated with cell–cell adhesion, receptor-mediated endocytosis, protein transport, cell surface receptor signaling pathway, integrin-mediated signaling pathway, membrane organization, and wound healing (**Figure 8D** and Supplementary Table S7). Of note, we observed KEGG enrichment associated with the PI3K-Akt signaling pathway, focal adhesion, Rap1 signaling, ECM-receptor interaction, and antigen processing and presentation (**Figure 8E** and Supplementary Table S8).

## Discussion

This study has provides the first evidence that EVs secreted by hAEC have a therapeutic potential for the treatment of liver fibrosis. Evidence of therapeutic effects of the secretome of MSC and other stem cells gave rise to a new theory, specifically that outcomes of cell therapies may be mediated by EVs (Tolar et al., 2010; Fouraschen et al., 2012; Lee et al., 2016). These vesicles, which play an important role in cell-to-cell communication, can alter the phenotype and fate of target cells (Xu et al., 2016). There is increasing evidence that exosomes influence physiological processes such as cell transformation (Greening et al., 2015a), immunoregulation (Greening et al., 2015b; Nawaz et al., 2016), and importantly, cancer progression (Atay et al., 2014; Melo et al., 2014), vaccination against infectious disease (Viaud et al., 2010), and vaccines for possible cancer treatments (Mignot et al., 2006; Gehrmann et al., 2013; Rak, 2013). These studies have led to several clinical and pre-clinical investigations of exosome/EV-based therapies (El Andaloussi et al., 2013; Buzas et al., 2014; Gyorgy et al., 2014; Vader et al., 2014; Xu et al., 2016). In the context of therapeutic applications, exosomes of selected cell types have been used as therapeutic agents in immune therapy, vaccination trials, regenerative medicine, and drug delivery (Fais et al., 2016). Exosomes also provide a largely unexplored source of diagnostic, prognostic, and predictive biomarkers (Rak, 2013; Nawaz et al., 2014, 2016; Greening et al., 2017). Interestingly, administration of EV derived from MSC resulted in reduced liver fibrosis (Li et al., 2012) and promoted hepatic regeneration (Tan et al., 2014).

In this study, we investigated the therapeutic efficacy of hAEC-CM, EV, and EVDM in a mouse model of CCl_4_-induced chronic liver fibrosis. We found that intravenous administration of CM, EV, and EVDM derived from hAEC significantly reduced HSC number and collagen production, reduced hepatic macrophage infiltration, and polarized macrophages to a pro-reparative phenotype *in vitro* and *in vivo*. Furthermore, only hAEC-EV significantly downregulated TGF-β1 signaling.

Carbon tetrachloride-induced liver fibrosis is a well-established model of liver injury (Manuelpillai et al., 2012). We commenced hAEC-CM, EV, and EVDM treatments while continuing CCl_4_ administration to model the persistent injury that occurs in the clinical setting and where clinical management would be relevant. During liver fibrosis, activation of HSC and subsequent transformation into myofibroblasts leads to the production of collagen and ECM accumulation. Activated HSC are widely measured by the expression of α-SMA (Lee Y.A. et al., 2015). Treatment with hAEC-CM, EV, and EVDM significantly reduced liver fibrosis, as shown by a reduction in the number of activated HSC and collagen proportionate area, even in the presence of continued CCl_4_ administration. We corroborated these findings *in vitro* using the human HSC cell line LX2, showing that hAEC-EV directly decreased collagen production in activated HSC. Interestingly, the therapeutic effect of hAEC-EV was similar to hAEC-CM, in regard to its reduction of liver fibrosis and HSC activation, which extends our previous findings showing that hAEC-CM contained soluble factors that have anti-fibrotic effects *in vitro* (Hodge et al., 2014). However, EVDM had a diminished ability to reduce collagen production *in vitro*, which is in contrast to our *in vivo* results. This could be a reflection of our use of an immortalized stellate cell line, which may not reflect an *in vivo* effect, as such the use of primary HSC should be the subject of further investigation. Alternatively, this may indicate that EVDM exerts its anti-fibrotic effects *in vivo* by acting on other cell types in the liver, rather than on stellate cells themselves.

TGF-β1 is well known to activate HSCs (Pradere et al., 2013). We found that hAEC-EV dramatically reduced the protein content of TGF-β1 in the livers of CCl_4_ mice; however, this was not achieved with hAEC-CM or EVDM. TGF-β was identified in both CM and EVDM and the dose in our study was 350 μl 3 times weekly for 4 weeks. A study by Huang et al. (2016) reported that MSC-CM 250 μl twice weekly for 3 weeks promoted therapeutic effects in a chronic liver fibrosis model. The higher dose in our study may explain the impaired ability of CM and EVDM to reduce hepatic TGF-β and indicates the importance of investigating the dose efficacy of CM and EVDM.

Hepatic macrophages are a heterogeneous population of cells that have a wide range of functions during homeostasis and disease (Lee Y.A. et al., 2015). Chronic liver fibrosis is associated with recruitment of macrophages that co-localize with fibrotic regions (Manuelpillai et al., 2012). Macrophage depletion using a transgenic mouse (CD11b-DTR) resulted in decreased fibrosis and HSC in chronic liver fibrosis induced by CCl_4_ (Duffield et al., 2005). In the present study, we found that CCl_4_-treated mice exhibited a significant increase in F4/80 positive macrophage infiltration, which was significantly decreased by hAEC-EV exposure.

Experimental evidence suggests that macrophages exert dual functions during liver fibrosis. The activation of macrophages during the injury phase is associated with ECM accumulation and HSC activation. On the other hand, macrophages activated during recovery resulted in matrix degradation (Duffield et al., 2005; Ramachandran et al., 2012). Phenotypic polarization from classically activated macrophages (M1) to alternatively activated macrophages (M2) is dependent on signals received from the local environment (Martinez and Gordon, 2014). M1 macrophages produce high levels of pro-inflammatory cytokines and are induced by LPS and interferon-γ (IFN-γ) (Martinez and Gordon, 2014). On the other hand, M2 macrophages produce anti-inflammatory cytokines, collagen-degrading enzymes and are induced by IL-4 and IL-13 (Song et al., 2000; López-Navarrete et al., 2011). Interestingly, the mannose and scavengers receptors present in M2 macrophages are able to phagocytose ECM and apoptotic cells leading to fibrosis resolution (López-Guisa et al., 2012; Wynn and Ramalingam, 2012). In this study, we used CD86 to identify M1 macrophages and CD206 for M2 macrophages (Bility et al., 2016). However, CD206 is expressed in M2 liver macrophages as well as liver sinusoidal endothelial cells (DeLeve, 2015). We therefore identified M1 macrophages by co-localization of CD86 and F4/80 while M2 macrophages were identified by co-localization of F4/80 and CD206. We found that hAEC-EV increased liver M2 macrophages in CCl_4_ mice, accompanied by a decrease in liver M1 macrophages, a similar effect seen with hAEC-CM and EVDM. This was corroborated *in vitro* using immortalized bone marrow macrophages. Taken together, our findings of reduced hepatic fibrosis area, reduced number of activated HSC and macrophages, reduced levels of TGF-β1 and polarization to M2 macrophages are consistent with those seen in our previous study, when we administered hAEC alone in the CCl_4_ mouse model (Manuelpillai et al., 2012). Data from our current study indicate that both the vesicular fraction and whole hAEC-CM may mediate the anti-fibrotic effects observed in CCl4-induced chronic liver fibrosis.

While the field of EV research has grown exponentially in recent years, findings from our current study indicate that the *soluble fraction* of secreted or shed cellular products should not be entirely disregarded. The proteomic analysis of CM, EV, and EVDM indicates the presence of proteins enriched for Rap1 pathway and PI3K/Akt pathway. Rap1 is involved in the control of cell proliferation and cell adhesion (Bos et al., 2001), while PI3K/Akt is implicated in macrophage polarization, cell cycle progression, and prevention of apoptosis (Chang et al., 2003; Vergadi et al., 2017). Therefore, our data suggest the PI3K/Akt pathway may modulate macrophage polarization. Interestingly, our proteomic analysis of hAEC-EV revealed the presence of proteins that target TGF-β signaling including MFGE8, HSP72, and SOD1. MFGE8 plays a critical role in reducing pulmonary fibrosis (Atabai et al., 2009). An et al. (2017) identified MFGE8 as an anti-fibrotic factor in the umbilical cord MSC secretome that inhibits TGF-β signaling and reduces liver fibrosis in mice. Moreover, HSP72 was found to attenuate renal tubulointerstitial fibrosis in obstructive nephropathy (Mao et al., 2008) and to inhibit epithelial-to-mesenchymal transition, which promotes collagen production, via effects on Smad2 activation (Zhou et al., 2010). Finally, the anti-fibrotic potential of SOD1 on radiation-induced fibrosis is mediated by downregulation of TGF-β signaling (Emerit et al., 2006). These proteins could play a role in the reduction of collagen production, fibrosis, and TGF-β expression observed in hAEC-EV-treated mice.

In summary, our findings suggest that the hAEC secretome, comprising soluble factors in hAEC-CM, both complete and EV depleted, in addition to hAEC-EV, had beneficial effects in reducing liver fibrosis in a murine model. This is the first study to provide evidence that hAEC-derived EVs can exert a therapeutic effect similar to what has been previously reported with hAEC in an experimental model of chronic liver fibrosis. Future studies could focus on identifying the specific anti-fibrotic factors that would support development of a clinically applicable therapy.

## Author Contributions

MA, DG, RL, and WS conceived and designed experiments. MA, JC, MZ, NK, RX, MS, AH, DG, RL, and WS performed experiments and analyzed data. MA, BL, DG, RL, and WS wrote the manuscript. All authors have read and approved the final manuscript.

## Conflict of Interest Statement

The authors declare that the research was conducted in the absence of any commercial or financial relationships that could be construed as a potential conflict of interest.

## Footnotes

1 www.uniprot.org

2 http://www.exocarta.org/

## Supplementary Material

FIGURE S1 Particle quantitation of hAEC-EVDM was performed by nanoparticle tracking analysis.

## References

[B1] Amin M. A. Sabry D. Rashed L. A. Aref W. M. el-Ghobary M. A. Farhan M. S. (2013). Short-term evaluation of autologous transplantation of bone marrow–derived mesenchymal stem cells in patients with cirrhosis: Egyptian study. *Clin. Transplant.* 27 607–612. 10.1111/ctr.12179 23923970

[B2] An S. Y. Jang Y. J. Lim H.-J. Han J. Lee J. Lee G. (2017). Milk fat globule-EGF factor 8, secreted by mesenchymal stem cells, protects against liver fibrosis in mice. *Gastroenterology* 152 1174–1186. 10.1053/j.gastro.2016.12.003 27956229

[B3] Atabai K. Jame S. Azhar N. Kuo A. Lam M. McKleroy W. (2009). Mfge8 diminishes the severity of tissue fibrosis in mice by binding and targeting collagen for uptake by macrophages. *J. Clin. Invest.* 119 3713–3722. 10.1172/JCI40053 19884654 PMC2786804

[B4] Atay S. Banskota S. Crow J. Sethi G. Rink L. Godwin A. K. (2014). Oncogenic KIT-containing exosomes increase gastrointestinal stromal tumor cell invasion. *Proc. Natl. Acad. Sci. U.S.A.* 111 711–716. 10.1073/pnas.1310501111 24379393 PMC3896203

[B5] Baglio S. R. Pegtel D. M. Baldini N. (2012). Mesenchymal stem cell secreted vesicles provide novel opportunities in (stem) cell-free therapy. *Front. Physiol.* 3:359. 10.3389/fphys.2012.00359 22973239 PMC3434369

[B6] Bansal R. Van Baarlen J. Storm G. Prakash J. (2015). The interplay of the Notch signaling in hepatic stellate cells and macrophages determines the fate of liver fibrogenesis. *Sci. Rep.* 5:18272. 10.1038/srep18272 26658360 PMC4677309

[B7] Bility M. T. Nio K. Li F. McGivern D. R. Lemon S. M. Feeney E. R. (2016). Chronic hepatitis C infection–induced liver fibrogenesis is associated with M2 macrophage activation. *Sci. Rep.* 6:39520. 10.1038/srep39520 28000758 PMC5175173

[B8] Bos J. L. de Rooij J. Reedquist K. A. (2001). Rap1 signalling: adhering to new models. *Nat. Rev. Mol. Cell Biol.* 2 369–377. 10.1038/35073073 11331911

[B9] Buzas E. I. Gyorgy B. Nagy G. Falus A. Gay S. (2014). Emerging role of extracellular vesicles in inflammatory diseases. *Nat. Rev. Rheumatol.* 10 356–364. 10.1038/nrrheum.2014.19 24535546

[B10] Chang F. Lee J. Navolanic P. Steelman L. Shelton J. Blalock W. (2003). Involvement of PI3K/Akt pathway in cell cycle progression, apoptosis, and neoplastic transformation: a target for cancer chemotherapy. *Leukemia* 17 590–603. 10.1038/sj.leu.2402824 12646949

[B11] Chang Y.-J. Liu J.-W. Lin P.-C. Sun L.-Y. Peng C.-W. Luo G.-H. (2009). Mesenchymal stem cells facilitate recovery from chemically induced liver damage and decrease liver fibrosis. *Life Sci.* 85 517–525. 10.1016/j.lfs.2009.08.003 19686763

[B12] Cho K. A. Lim G. W. Joo S. Y. Woo S. Y. Seoh J. Y. Cho S. J. (2011). Transplantation of bone marrow cells reduces CCl4-induced liver fibrosis in mice. *Liver Int.* 31 932–939. 10.1111/j.1478-3231.2010.02364.x 21092070

[B13] Cox J. Mann M. (2008). MaxQuant enables high peptide identification rates, individualized p.p.b.-range mass accuracies and proteome-wide protein quantification. *Nat. Biotechnol.* 26 1367–1372. 10.1038/nbt.1511 19029910

[B14] DeLeve L. D. (2015). Liver sinusoidal endothelial cells in hepatic fibrosis. *Hepatology* 61 1740–1746. 10.1002/hep.27376 25131509 PMC4333127

[B15] Duffield J. S. Forbes S. J. Constandinou C. M. Clay S. Partolina M. Vuthoori S. (2005). Selective depletion of macrophages reveals distinct, opposing roles during liver injury and repair. *J. Clin. Invest.* 115 56–65. 10.1172/JCI200522675 15630444 PMC539199

[B16] El Andaloussi S. Mager I. Breakefield X. O. Wood M. J. (2013). Extracellular vesicles: biology and emerging therapeutic opportunities. *Nat. Rev. Drug Discov.* 12 347–357. 10.1038/nrd3978 23584393

[B17] Emerit J. Samuel D. Pavio N. (2006). Cu–Zn super oxide dismutase as a potential antifibrotic drug for hepatitis C related fibrosis. *Biomed. Pharmacother.* 60 1–4. 10.1016/j.biopha.2005.09.002 16297593

[B18] Fais S. O’Driscoll L. Borras F. E. Buzas E. Camussi G. Cappello F. (2016). Evidence-based clinical use of nanoscale extracellular vesicles in nanomedicine. *ACS Nano* 10 3886–3899. 10.1021/acsnano.5b08015 26978483

[B19] Fouraschen S. M. Pan Q. de Ruiter P. E. Farid W. R. Kazemier G. Kwekkeboom J. (2012). Secreted factors of human liver-derived mesenchymal stem cells promote liver regeneration early after partial hepatectomy. *Stem Cells Dev.* 21 2410–2419. 10.1089/scd.2011.0560 22455365

[B20] Francoz C. Belghiti J. Durand F. (2007). Indications of liver transplantation in patients with complications of cirrhosis. *Best Pract. Res. Clin. Gastroenterol.* 21 175–190. 10.1016/j.bpg.2006.07.007 17223504

[B21] Gehrmann U. Hiltbrunner S. Georgoudaki A. M. Karlsson M. C. Naslund T. I. Gabrielsson S. (2013). Synergistic induction of adaptive antitumor immunity by codelivery of antigen with alpha-galactosylceramide on exosomes. *Cancer Res.* 73 3865–3876. 10.1158/0008-5472.CAN-12-3918 23658368

[B22] Gopal S. K. Greening D. W. Mathias R. A. Ji H. Rai A. Chen M. (2015). YBX1/YB-1 induces partial EMT and tumourigenicity through secretion of angiogenic factors into the extracellular microenvironment. *Oncotarget* 6 13718–13730. 10.18632/oncotarget.3764 25980435 PMC4537044

[B23] Gorshkov V. Verano-Braga T. Kjeldsen F. (2015). SuperQuant: a data processing approach to increase quantitative proteome coverage. *Anal. Chem.* 87 6319–6327. 10.1021/acs.analchem.5b01166 25978296

[B24] Greening D. W. Gopal S. K. Mathias R. A. Liu L. Sheng J. Zhu H. J. (2015a). Emerging roles of exosomes during epithelial-mesenchymal transition and cancer progression. *Semin. Cell Dev. Biol.* 40 60–71. 10.1016/j.semcdb.2015.02.008 25721809

[B25] Greening D. W. Gopal S. K. Xu R. Simpson R. J. Chen W. (2015b). Exosomes and their roles in immune regulation and cancer. *Semin. Cell Dev. Biol.* 40 72–81. 10.1016/j.semcdb.2015.02.009 25724562

[B26] Greening D. W. Ji H. Chen M. Robinson B. W. S. Dick I. M. Creaney J. (2016a). Secreted primary human malignant mesothelioma exosome signature reflects oncogenic cargo. *Sci. Rep.* 6:32643. 10.1038/srep32643 27605433 PMC5015102

[B27] Greening D. W. Nguyen H. Elgass K. Simpson R. J. Salamonsen L. A. (2016b). Human endometrial exosomes contain hormone-specific cargo modulating trophoblast adhesive capacity: insights into endometrial-embryo interactions. *Biol. Reprod.* 94 1–15. 10.1095/biolreprod.115.134890 26764347

[B28] Greening D. W. Xu R. Gopal S. K. Rai A. Simpson R. J. (2017). Proteomic insights into extracellular vesicle biology - defining exosomes and shed microvesicles. *Expert Rev. Proteomics* 14 69–95. 10.1080/14789450.2017.1260450 27838931

[B29] Gyorgy B. Hung M. E. Breakefield X. O. Leonard J. N. (2014). Therapeutic applications of extracellular vesicles: clinical promise and open questions. *Annu. Rev. Pharmacol. Toxicol.* 55 439–464. 10.1146/annurev-pharmtox-010814-124630 25292428 PMC4445965

[B30] Hodge A. Lourensz D. Vaghjiani V. Nguyen H. Tchongue J. Wang B. (2014). Soluble factors derived from human amniotic epithelial cells suppress collagen production in human hepatic stellate cells. *Cytotherapy* 16 1132–1144. 10.1016/j.jcyt.2014.01.005 24642017

[B31] Huang B. Cheng X. Wang H. Huang W. Wang D. Zhang K. (2016). Mesenchymal stem cells and their secreted molecules predominantly ameliorate fulminant hepatic failure and chronic liver fibrosis in mice respectively. *J. Transl. Med.* 14:1. 10.1186/s12967-016-0792-1 26861623 PMC4746907

[B32] Huang C. K. Lee S. O. Lai K. P. Ma W. L. Lin T. H. Tsai M. Y. (2013). Targeting androgen receptor in bone marrow mesenchymal stem cells leads to better transplantation therapy efficacy in liver cirrhosis. *Hepatology* 57 1550–1563. 10.1002/hep.26135 23150236

[B33] Huang da W. Sherman B. T. Lempicki R. A. (2009). Systematic and integrative analysis of large gene lists using DAVID bioinformatics resources. *Nat. Protoc.* 4 44–57. 10.1038/nprot.2008.211 19131956

[B34] Lee S. C. Jeong H. J. Lee S. K. Kim S.-J. (2016). Hypoxic conditioned medium from human adipose-derived stem cells promotes mouse liver regeneration through JAK/STAT3 Signaling. *Stem Cells Transl. Med.* 5 816–825. 10.5966/sctm.2015-0191 27102647 PMC4878330

[B35] Lee S. K. Lee S. C. Kim S.-J. (2015). A novel cell-free strategy for promoting mouse liver regeneration: utilization of a conditioned medium from adipose-derived stem cells. *Hepatol. Int.* 9 310–320. 10.1007/s12072-014-9599-4 25788187

[B36] Lee Y. A. Wallace M. C. Friedman S. L. (2015). Pathobiology of liver fibrosis: a translational success story. *Gut* 64 830–841. 10.1136/gutjnl-2014-306842 25681399 PMC4477794

[B37] Li T. Yan Y. Wang B. Qian H. Zhang X. Shen L. (2012). Exosomes derived from human umbilical cord mesenchymal stem cells alleviate liver fibrosis. *Stem Cells Dev.* 22 845–854. 10.1089/scd.2012.0395 23002959 PMC3585469

[B38] Liedtke C. Luedde T. Sauerbruch T. Scholten D. Streetz K. Tacke F. (2013). Experimental liver fibrosis research: update on animal models, legal issues and translational aspects. *Fibrogenesis Tissue Repair* 6:19. 10.1186/1755-1536-6-19 24274743 PMC3850878

[B39] López-Guisa J. M. Cai X. Collins S. J. Yamaguchi I. Okamura D. M. Bugge T. H. (2012). Mannose receptor 2 attenuates renal fibrosis. *J. Am. Soc. Nephrol.* 23 236–251. 10.1681/ASN.2011030310 22095946 PMC3269177

[B40] López-Navarrete G. Ramos-Martínez E. Suárez-Álvarez K. Aguirre-García J. Ledezma-Soto Y. León-Cabrera S. (2011). Th2-associated alternative Kupffer cell activation promotes liver fibrosis without inducing local inflammation. *Int. J. Biol. Sci.* 7 1273–1286. 10.7150/ijbs.7.1273 22110380 PMC3221364

[B41] Lötvall J. Hill A. F. Hochberg F. Buzás E. I. Di Vizio D. Gardiner C. (2014). Minimal experimental requirements for definition of extracellular vesicles and their functions: a position statement from the international society for extracellular vesicles. *J. Extracell. Vesicles* 3:26913. 10.3402/jev.v3.26913 25536934 PMC4275645

[B42] Luber C. A. Cox J. Lauterbach H. Fancke B. Selbach M. Tschopp J. (2010). Quantitative proteomics reveals subset-specific viral recognition in dendritic cells. *Immunity* 32 279–289. 10.1016/j.immuni.2010.01.013 20171123

[B43] Makridakis M. Roubelakis M. G. Vlahou A. (2013). Stem cells: insights into the secretome. *Biochim. Biophys. Acta* 1834 2380–2384. 10.1016/j.bbapap.2013.01.032 23376432

[B44] Manuelpillai U. Lourensz D. Vaghjiani V. Tchongue J. Lacey D. Tee J.-Y. (2012). Human amniotic epithelial cell transplantation induces markers of alternative macrophage activation and reduces established hepatic fibrosis. *PLOS ONE* 7:e38631. 10.1371/journal.pone.0038631 22719909 PMC3375296

[B45] Manuelpillai U. Tchongue J. Lourensz D. Vaghjiani V. Samuel C. S. Liu A. (2010). Transplantation of human amnion epithelial cells reduces hepatic fibrosis in immunocompetent CCl4-treated mice. *Cell Transplant.* 19 1157–1168. 10.3727/096368910X504496 20447339

[B46] Mao H. Li Z. Zhou Y. Li Z. Zhuang S. An X. (2008). HSP72 attenuates renal tubular cell apoptosis and interstitial fibrosis in obstructive nephropathy. *Am. J. Physiol. Renal Physiol.* 295 F202–F214. 10.1152/ajprenal.00468.2007 18417540 PMC4116425

[B47] Martinez F. O. Gordon S. (2014). The M1 and M2 paradigm of macrophage activation: time for reassessment. *F1000Prime Rep.* 6:13. 10.12703/P6-13 24669294 PMC3944738

[B48] Meldolesi J. (2015). “Exosomes, ectosomes and the two together. physiology and pathology,” in *Forum on Immunopathological Diseases and Therapeutics*, eds Bonavida B. Atassi M. Z. (Danbury, CT: Begell House Inc).

[B49] Melo S. Sugimoto H. O’Connell J. Kato N. Villanueva A. Vidal A. (2014). Cancer exosomes perform cell-independent MicroRNA biogenesis and promote tumorigenesis. *Cancer Cell* 26 707–721. 10.1016/j.ccell.2014.09.005 25446899 PMC4254633

[B50] Mignot G. Roux S. Thery C. Segura E. Zitvogel L. (2006). Prospects for exosomes in immunotherapy of cancer. *J. Cell Mol. Med.* 10 376–388. 10.1111/j.1582-4934.2006.tb00406.x 16796806 PMC3933128

[B51] Mohamadnejad M. Alimoghaddam K. Bagheri M. Ashrafi M. Abdollahzadeh L. Akhlaghpoor S. (2013). Randomized placebo - controlled trial of mesenchymal stem cell transplantation in decompensated cirrhosis. *Liver Int.* 33 1490–1496. 10.1111/liv.12228 23763455

[B52] Murphy S. Lim R. Dickinson H. Acharya R. Rosli S. Jenkin G. (2011). Human amnion epithelial cells prevent bleomycin-induced lung injury and preserve lung function. *Cell Transplant.* 20 909–923. 10.3727/096368910X543385 21092408

[B53] Murphy S. Rosli S. Acharya R. Mathias L. Lim R. Wallace E. (2010). Amnion epithelial cell isolation and characterization for clinical use. *Curr. Protoc. Stem Cell Biol.* 13 1E.6.1–1E.6.25. 10.1002/9780470151808.sc01e06s13 20373516

[B54] Nawaz M. Camussi G. Valadi H. Nazarenko I. Ekstrom K. Wang X. (2014). The emerging role of extracellular vesicles as biomarkers for urogenital cancers. *Nat. Rev. Urol.* 11 688–701. 10.1038/nrurol.2014.301 25403245

[B55] Nawaz M. Fatima F. Nazarenko I. Ekstrom K. Murtaza I. Anees M. (2016). Extracellular vesicles in ovarian cancer: applications to tumor biology, immunotherapy and biomarker discovery. *Exp. Rev. Proteom.* 13 395–409. 10.1586/14789450.2016.1165613 26973172

[B56] Ng Y. H. Rome S. Jalabert A. Forterre A. Singh H. Hincks C. L. (2013). Endometrial exosomes/microvesicles in the uterine microenvironment: a new paradigm for embryo-endometrial cross talk at implantation. *PLOS ONE* 8:e58502. 10.1371/journal.pone.0058502 23516492 PMC3596344

[B57] Pradere J. P. Kluwe J. Minicis S. Jiao J. J. Gwak G. Y. Dapito D. H. (2013). Hepatic macrophages but not dendritic cells contribute to liver fibrosis by promoting the survival of activated hepatic stellate cells in mice. *Hepatology* 58 1461–1473. 10.1002/hep.26429 23553591 PMC3848418

[B58] Pratama G. Vaghjiani V. Tee J. Y. Liu Y. H. Chan J. Tan C. (2011). Changes in culture expanded human amniotic epithelial cells: implications for potential therapeutic applications. *PLOS ONE* 6:e26136. 10.1371/journal.pone.0026136 22073147 PMC3206797

[B59] Rak J. (2013). Extracellular vesicles - biomarkers and effectors of the cellular interactome in cancer. *Front. Pharmacol.* 4:21. 10.3389/fphar.2013.00021 23508692 PMC3589665

[B60] Ramachandran P. Pellicoro A. Vernon M. A. Boulter L. Aucott R. L. Ali A. (2012). Differential Ly-6C expression identifies the recruited macrophage phenotype, which orchestrates the regression of murine liver fibrosis. *Proc. Natl. Acad. Sci. U.S.A.* 109 E3186–E3195. 10.1073/pnas.1119964109 23100531 PMC3503234

[B61] Song E. Ouyang N. Hörbelt M. Antus B. Wang M. Exton M. S. (2000). Influence of alternatively and classically activated macrophages on fibrogenic activities of human fibroblasts. *Cell Immunol.* 204 19–28. 10.1006/cimm.2000.1687 11006014

[B62] Tan C. Y. Lai R. C. Wong W. Dan Y. Y. Lim S.-K. Ho H. K. (2014). Mesenchymal stem cell-derived exosomes promote hepatic regeneration in drug-induced liver injury models. *Stem Cell Res. Ther.* 5:76. 10.1186/scrt465 24915963 PMC4229780

[B63] Tauro B. J. Greening D. W. Mathias R. A. Ji H. Mathivanan S. Scott A. M. (2012a). Comparison of ultracentrifugation, density gradient separation, and immunoaffinity capture methods for isolating human colon cancer cell line LIM1863-derived exosomes. *Methods* 56 293–304. 10.1016/j.ymeth.2012.01.002 22285593

[B64] Tauro B. J. Greening D. W. Mathias R. A. Mathivanan S. Ji H. Simpson R. J. (2012b). Two distinct populations of exosomes are released from LIM1863 colon carcinoma cell-derived organoids. *Mol. Cell. Proteom.* 12 587–598. 10.1074/mcp.M112.021303 23230278 PMC3591653

[B65] Tolar J. Le Blanc K. Keating A. Blazar B. R. (2010). Concise review: hitting the right spot with mesenchymal stromal cells. *Stem Cells* 28 1446–1455. 10.1002/stem.459 20597105 PMC3638893

[B66] Vader P. Breakefield X. O. Wood M. J. (2014). Extracellular vesicles: emerging targets for cancer therapy. *Trends Mol. Med.* 20 385–393. 10.1016/j.molmed.2014.03.002 24703619 PMC4082760

[B67] Vergadi E. Ieronymaki E. Lyroni K. Vaporidi K. Tsatsanis C. (2017). Akt signaling pathway in macrophage activation and M1/M2 polarization. *J. Immunol.* 198 1006–1014. 10.4049/jimmunol.1601515 28115590

[B68] Viaud S. Thery C. Ploix S. Tursz T. Lapierre V. Lantz O. (2010). Dendritic cell-derived exosomes for cancer immunotherapy: what’s next? *Cancer Res.* 70 1281–1285. 10.1158/0008-5472.CAN-09-3276 20145139

[B69] Vosdoganes P. Hodges R. J. Lim R. Westover A. J. Acharya R. Y. Wallace E. M. (2011). Human amnion epithelial cells as a treatment for inflammation-induced fetal lung injury in sheep. *Am. J. Obstet. Gynecol.* 205 156.e26–33. 10.1016/j.ajog.2011.03.054 21640967

[B70] Vosdoganes P. Lim R. Koulaeva E. Chan S. T. Acharya R. Moss T. J. (2013). Human amnion epithelial cells modulate hyperoxia-induced neonatal lung injury in mice. *Cytotherapy* 15 1021–1029. 10.1016/j.jcyt.2013.03.004 23643416

[B71] Wolbank S. Peterbauer A. Fahrner M. Hennerbichler S. Van Griensven M. Stadler G. (2007). Dose-dependent immunomodulatory effect of human stem cells from amniotic membrane: a comparison with human mesenchymal stem cells from adipose tissue. *Tissue Eng.* 13 1173–1183. 10.1089/ten.2006.0313 17518752

[B72] Wynn T. A. Ramalingam T. R. (2012). Mechanisms of fibrosis: therapeutic translation for fibrotic disease. *Nat. Med.* 18 1028–1040. 10.1038/nm.2807 22772564 PMC3405917

[B73] Xu R. Greening D. W. Zhu H.-J. Takahashi N. Simpson R. J. (2016). Extracellular vesicle isolation and characterization: toward clinical application. *J. Clin. Invest.* 126 1152–1162. 10.1172/JCI81129 27035807 PMC4811150

[B74] Zhou Y. Mao H. Li S. Cao S. Li Z. Zhuang S. (2010). HSP72 inhibits Smad3 activation and nuclear translocation in renal epithelial-to-mesenchymal transition. *J. Am. Soc. Nephrol.* 21 598–609. 10.1681/ASN.2009050552 20133478 PMC2844311

