# Supplementary figures and images for: The Human Amnion Epithelial Cell Secretome Decreases Hepatic Fibrosis in Mice with Chronic Liver Fibrosis
Source: Front Pharmacol. 2017 Oct 24;8:748. doi: 10.3389/fphar.2017.00748 (PMC5660722; doi:10.3389/fphar.2017.00748)

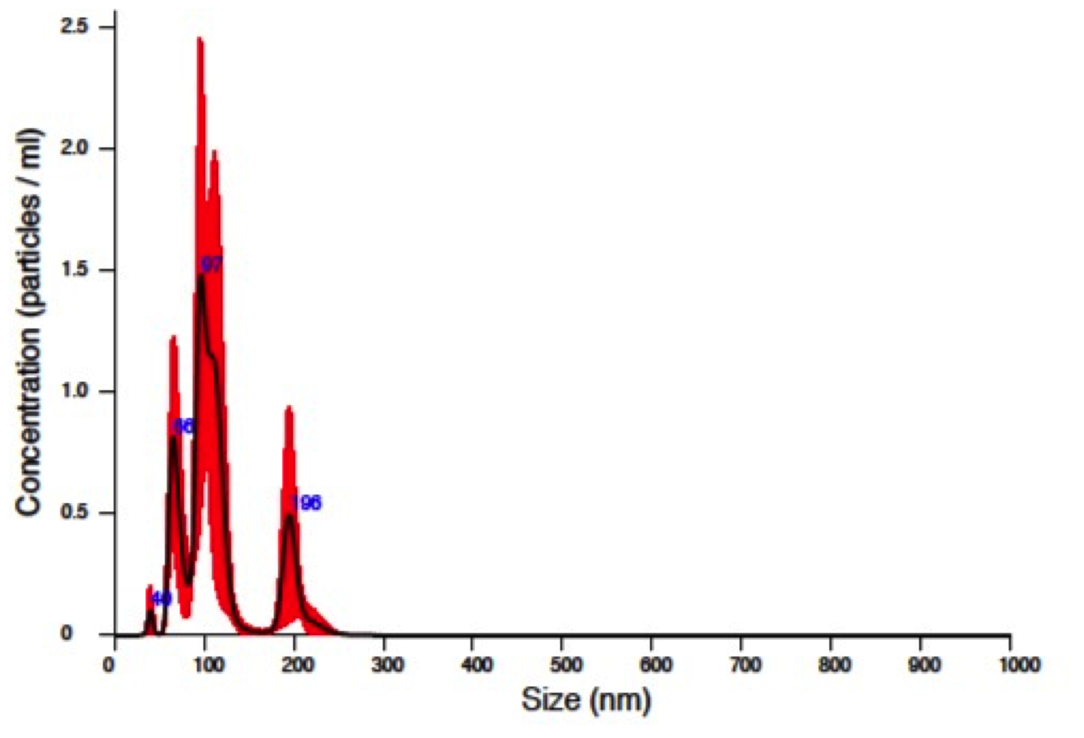

Supplement: FIGURE S1 — Particle quantitation of hAEC-EVDM was performed by nanoparticle tracking analysis. [file Image_1.TIFF]
